# Strain Engineering to Modify the Electrochemistry of Energy Storage Electrodes

**DOI:** 10.1038/srep27542

**Published:** 2016-06-10

**Authors:** Nitin Muralidharan, Rachel Carter, Landon Oakes, Adam P. Cohn, Cary L. Pint

**Affiliations:** 1 Interdisciplinary Materials Science Program, Vanderbilt University, Nashville, TN 37235 USA; 2 Department of Mechanical Engineering, Vanderbilt University, Nashville, TN 37235 USA

## Abstract

Strain engineering has been a critical aspect of device design in semiconductor manufacturing for the past decade, but remains relatively unexplored for other applications, such as energy storage. Using mechanical strain as an input parameter to modulate electrochemical potentials of metal oxides opens new opportunities intersecting fields of electrochemistry and mechanics. Here we demonstrate that less than 0.1% strain on a Ni-Ti-O based metal-oxide formed on superelastic shape memory NiTi alloys leads to anodic and cathodic peak potential shifts by up to ~30 mV in an electrochemical cell. Moreover, using the superelastic properties of NiTi to enable strain recovery also recovers the electrochemical potential of the metal oxide, providing mechanistic evidence of strain-modified electrochemistry. These results indicate that mechanical energy can be coupled with electrochemical systems to efficiently design and optimize a new class of strain-modulated energy storage materials.

Traditional routes to design materials for electrochemical applications require modification of material chemical composition to control reduction-oxidation energetics when coupled with an electrolyte^1^,^2^. This causes the search for improved nanomaterials in electrochemical applications to be driven by discovery-focused nanomaterial synthesis and fabrication. Due to the complex cooperative nature of energy storage device performance based on the pairing of electrodes and electrolytes, such routes rarely lead to new materials with characteristics, such as operating voltage, that outperform existing materials. Further, whereas computational guidance has brought about a new paradigm to predict targeted material compounds that can improve advanced energy storage systems^3^,^4^, experimentalists often remain challenged by the synthesis process of such compounds, many of which are not naturally occurring. This presents a fundamental bottleneck in the conventional approach in which electrochemistry-oriented material research and development occurs that limits the rate of industry innovation in energy storage (and conversion) systems.

Strain engineering, a concept widely implemented in semiconductor electronics^5^,^6^, opens new opportunities to engineer materials for electrochemical systems. The six-dimensional parameter space of the strain tensor^7^ enables a nanostructure with a fixed chemical composition to have electronic and physical properties finely modulated in a manner that is virtually impossible to replicate by varying chemical composition. Unlike bulk materials, many nanostructures exhibit sizes where strain can homogenously propagate throughout the whole material, instead of only on a surface or an interface^6^,^8^,^9^,^10^,^11^,^12^,^13^,^14^. In this spirit, recent efforts have demonstrated the capability of strain in nanostructures to modify the energy landscape of catalytic and electro-catalytic surface-bound reactions^15^,^16^,^17^,^18^,^19^ and modify oxygen ion diffusion in fuel cell technology^20^,^21^,^22^. The direct controlled correlation between mechanical strain as an input parameter and electrochemical processes in nanostructures for energy harvesting applications has only very recently been reported^23^.

In the specific case of energy storage electrodes such as pseudocapacitors and batteries^1^,^24^, Faradaic reactions especially in metal oxides induce a change to the oxidation state of the active material often regulated by the physical characteristics of the lattice structure to enable insertion or alloying of an ion species. Focus of current research efforts so far have been on the adverse effects of strain arising from the changes to the host lattice structures during electrochemical cycling of energy storage electrodes^25^,^26^,^27^. In this regard, mechanical strain imposed onto a nanostructure leading to both physical and electronic changes that can synergistically influence energy storage redox reactions has not yet been reported despite significant advances in the ability to produce, image, and understand strain effects in materials^15^,^28^, especially those related to semiconductor electronics. Theoretical studies have recently highlighted the prospect of pre-straining materials^29^ even though experimental efforts in this direction remain elusive.

Here we demonstrate a study where nanostructured metal oxide materials are synthesized directly on the surface of a superelastic/shapememory NiTi wire. Under the application of strain to the NiTi and transferred to the surface-bound oxides, we observe consistent shifts in the anodic and cathodic potentials. By recovering this imposed strain, we observe consistent recovery of the electrochemical potentials, clearly demonstrating that strain, as opposed to other effects, is modulating the shifts in electrochemical potentials and can be a viable tool for the design of energy storage materials.

## Metal-oxide nanostructures on superelastic NiTi

**Figure 1:**
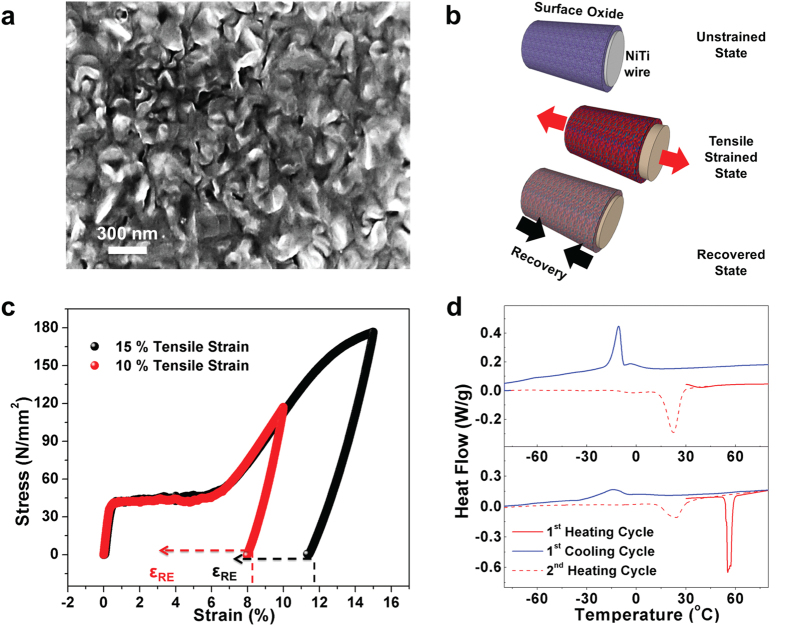
Strain engineering the surface oxide using NiTi alloy. (**a**) SEM image of the active NiO-TiO_2_ based metal oxide formed on the surface of the NiTi alloy. (**b**) Schematic illustration of experimental system, including the application of strain and strain recovery on a NiTi wire with surface-bound active material. (**c**) Stress-strain behavior of the NiTi superelastic alloy deformed up to 10% and 15% tensile strain. The dashed lines and ε_RE_ indicates the heat assisted transformation process and the recovered strain respectively. (**d**) DSC thermograms of the unstrained (0%) and the tensile strained (15%) states. Red (line and dots) represents the heating cycle and blue line represents the cooling cycles.

A Ni-Ti-O based oxide was grown on NiTi superelastic wires at 600 °C comprising of a mixed NiO-TiO_2_ layer formed on top of a titanate layer on the surface of the NiTi material^30^,^31^. EDS maps (supporting information) obtained from the surface of the wire indicated a mixed Ni-Ti-O based surface oxide. Scanning electron microscopy (SEM) (Fig. 1a) was performed on the nanostructured surface oxides formed on the wire. To study strain-related modifications to energy storage processes, NiTi wires were tensile deformed to 10% and 15% strain at room temperature using an Instron mechanical testing system, with a corresponding small percentage of this input strain (less than 1%) transferred to the surface-bound oxide nanomaterial. The strained and unstrained wires with the mixed oxide layers were sonicated in peroxide at room temperature to impart nanotexturing to the oxide. This leads to a material architecture where nanostructured petals of NiO-TiO_2_-based metal oxides are conformally coated on the surface of a NiTi wire in a seamless manner (Fig. 1b). The Ni-Ti-O surface metal oxide was further examined through Transmission electron microscopy (TEM) (supporting information, Figure S5) indicating the crystalline nanostructure of the surface oxide. The presence of the constituent elements of the nanostructured oxide (nickel, titanium, and oxygen) was further verified using STEM EDS maps. This architecture is ideally suited to correlate strain as an input parameter to the NiTi to assess its effect on the surface-bound redox active Ni-Ti-O material for energy storage applications.

The stress-strain response of the alloy is shown in Fig. 1c. The deformation behavior of the wire is described by elastic deformation of the austenite followed by the stress induced martensitic transformation below and up to 5% strain. Beyond 5% strain, oriented martensites begin to deform leading to plastic deformation of oriented martensites above 15% strain. These stress induced martensites can be transformed back to austenite by heating strained wires beyond their austenitic finish (A_f_) temperatures. The final mechanical strain input onto the NiTi alloy is ~8% and 11% for tensile deformations of 10% and 15% respectively, due to the intrinsic mechanical recovery of the alloy^32^. Strain recovery (10% R, 15% R) in the alloy was also measured to be ~4.5% and 3.5% respectively.

Differential scanning calorimetry (DSC) thermograms on 15% deformed and 0% deformed wires (Fig. 1d) demonstrates the transformation from stress induced martensite to parent austenite during the first heating cycle is present for the 15% tensile deformed wire in the temperature range of 50 to 60 °C whereas the unstrained (0%) wire showed no such transformations in this temperature range. This transformation during the first heating cycle is complete at a temperature of 60 °C which is the austenitic finish temperature (A_f_). Based on the DSC results, the 15% and 10% tensile deformed alloy was heated to 60 °C in vacuum to complete the reverse transformation from stress induced martensitic state to parent austenite state. The transformation temperature around 60 °C is ideal in the case of metal oxides to avoid annealing effects which are prevalent at higher temperatures.

## Spectroscopic strain analysis

**Figure 2:**
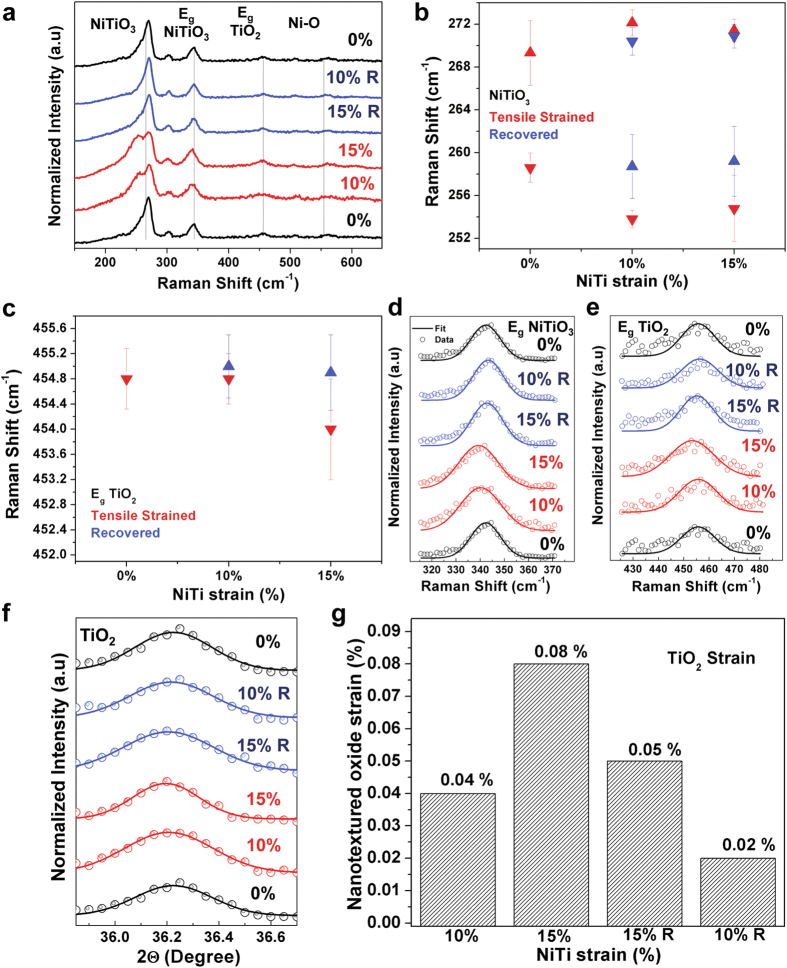
Characterizing strain on redox active nanostructures. (**a**) Raman spectra of the strained (10% and 15%), unstrained (0%) and recovered (10% R and 15% R) states. (**b,c**) Raman maps based on 800**–**1000 individual scans showing average strain effect on titanate-NiTiO_3_ (**b**) and TiO_2_ (**c**) active materials. (**d,e**) Selected spectra and the fitted curves of (**d**) E_g_ mode of NiTiO_3_ and (**e**) E_g_ mode of TiO_2_ at various strained and recovered states. (**f**) Selected X-Ray diffraction spectra and Gaussian fits of the peak corresponding to (012) plane of TiO_2_ (brookite) at various strained and recovered states. (**g**) Percent strain corresponding to (**f** ) based on both strained and recovered states. Note classifications of 10% and 15% strain correspond to strain applied to NiTi only.

As we use mechanical strain as an input parameter on NiTi to transfer strain to a surface oxide active material, XRD and Raman spectroscopy provide insight into strain transfer that enables controlled assessment of strain effects on electrochemical measurements. To characterize the transfer of strain applied to the superelastic/shapememory NiTi to the metal oxide nanostructured active material on the surface, Raman spectroscopy was carried out with 532 nm excitations (Fig. 2). The coupling of strain into a material will modify local stretch modes, hence enabling Raman spectroscopy as a sensitive tool for identifying strain in materials with distinct Raman modes. Similar to previous reports on heat treated NiTi alloys^33^ we find the Raman spectra of the nanostructured surface oxide to exhibit a strong peak near 269 cm^−1^ attributed to the titanate mode which are the Raman active modes of NiTiO_3_^34^,^35^,^36^,^37^, peaks centered on 300 cm^−1^ and 342 cm^−1^ attributed to the E_g_ modes of NiTiO_3_^38^,^39^,^40^,^41^, and a peak near 454 cm^−1^ attributed to the E_g_ mode of rutile phase TiO_2_^42^,^43^. Whereas shifts and mode-splitting can be observed in these peaks as a function of applied strain, statistical Raman maps over large areas of the surface (800–1000 total Raman scans in each map) were performed to quantify strain-related shifts observed in the active materials, specifically for the modes identified in Fig. 2a. Lorentzian fits were applied to these Raman modes (Fig. 2b,c) and statistically validated strain effects were isolated. Upon strain recovery, these Raman modes revert back near or toward the unstrained peak positions, indicating the correlation between the measured Raman response and strain applied on the petaled nanostructured oxide surface. Importantly, the shift and reversal of the Raman modes upon strain and strain recovery across a large-area statistical sampling of the oxide without a significant bimodal or broadening effect on the peak distributions indicates the absence of interfacial delamination as a stress relief mechanism in this material system. Furthermore, the *E*_*g*_ mode of NiTiO_3_ (Fig. 2d) and TiO_2_ (Fig. 2e) are observed to exhibit similar recoverable shifts in the Raman modes that can be correlated with strain and straightforwardly identified. The low intensities of the observed Ni-O stretch modes^44^,^45^,^46^ near 508 cm^−1^ and 571 cm^−1^ prohibited quantitative analysis on these modes.

Despite the clear signature of strain deduced through Raman spectroscopy, X-ray diffraction (XRD) provides further quantitative insight. XRD measurements indicate the same trend as Raman spectroscopy, where shifts toward lower angles are observed upon strain application, with recovery leading to the opposite shift. This is specifically shown for the (012) plane of TiO_2_ (Brookite) in Fig. 2f,g. To accurately determine the strain on the surface oxide using X-Ray diffractograms, Gaussian fits were applied to the obtained peaks. A standard analytical procedure was used to correlate strain to the *d*-spacing of the strained, unstrained, and recovered states given by the following equation,




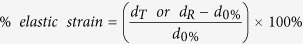




where, *d*_*T*_, *d*_*R*_ and *d*_*0%*_ represent the *d*-spacing of the tensile strained states, the recovered states and the unstrained state, respectively. This enables the strain experienced by the surface oxide to be accurately determined. The application of 10% and 15% strain to the NiTi alloy is observed to transfer ~0.04% and ~0.08% strain to the surface oxide, which can also be recovered as described in Fig. 1c. Full XRD analysis of the material is discussed in the supporting information. Strain transfer using NiTi superelastic/shapememory alloys have so far been limited to metals^17^ and alloys deposited on the surface with a maximum strain transfer of 2.18% achieved for an Fe-Pt metal alloy^47^,^48^. The surface oxide on the NiTi alloy, being a brittle ceramic is not as ductile as metals and metal alloys, therefore experiences cracking with less than 1% applied strain^49^. Overall, strain measured at <0.1% is expected due to the stress relief through cracking of the surface oxide and strain transfer across a nanostructured-bulk interface. The oxide layer will crack until reaching a critical tensile strain where the crack density saturates and strain is further transferred to cracked islands to ”lock in” elastic strain on the surface oxide^50^. This critical tensile strain for crack density saturation is modulated by both the thickness of the oxide layer as well as morphology of the nanostructured surface oxide. As 10% and 15% strain applied to the NiTi alloy exceeds the critical tensile strain for the thick oxide layer these measurements are carried out in a regime where increased strain on the NiTi will lead to increased strain on the oxide layer, which can be experimentally studied through Raman and XRD measurements. Moreover, XRD and Raman spectroscopy provide a combined toolset that can together identify the signature of elastic strain locked in a metal or metal oxide crystal structure to impact electrochemical behavior^17^. Combined with the versatility of superelastic/shape memory NiTi materials, this provides the ideal platform for assessing and understanding the mechano-electrochemical response of the surface oxide.

## Strain engineered electrochemistry

**Figure 3:**
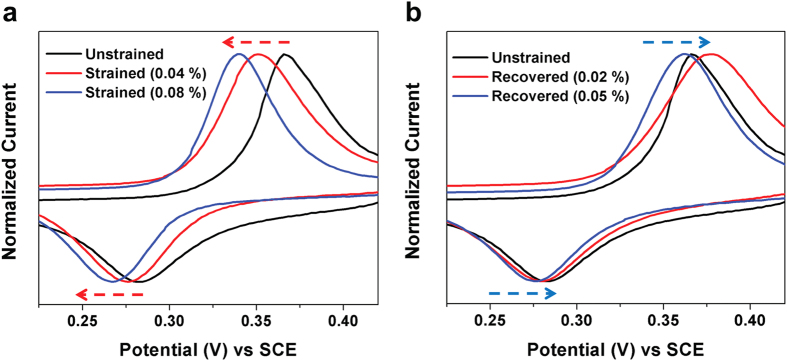
Correlating strain to electrochemical response. (**a**) Cyclic voltammograms with normalized current densities of the unstrained and the tensile strained (0.04%) and (0.08%) states based on surface oxides at a voltage window of 0.2 to 0.45 V. (**b**) Cyclic voltammograms with normalized current densities of the unstrained and recovered (0.02%) and (0.05%) states based on surface oxides at a voltage window of 0.2 to 0.45 V at scan rate of 100 mV/s.

**Figure 4:**
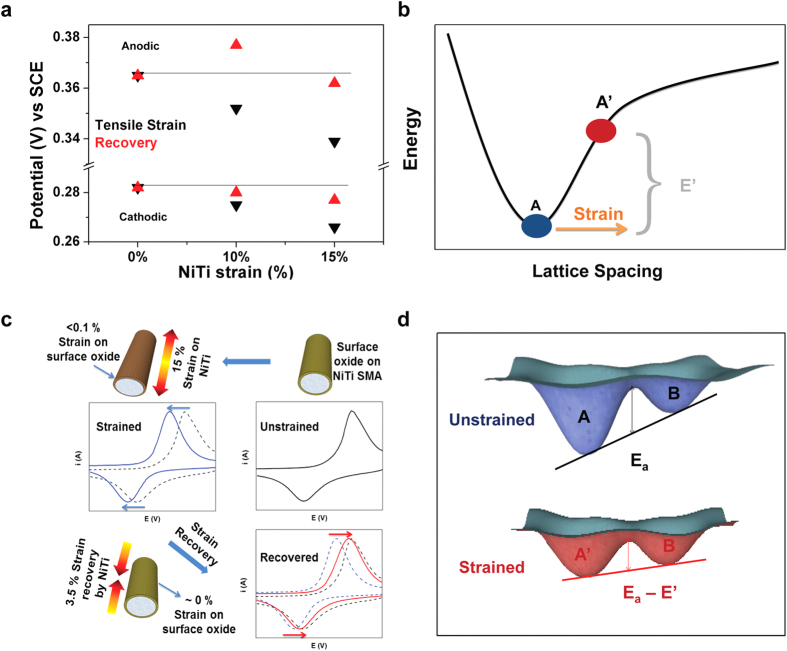
The role of strain to modify energy storage electrochemistry. (**a**) Anodic and cathodic peak potentials plotted versus SCE at various unstrained, strained, and recovered states of the NiTi alloy. (**b**) General plot of the total cohesive energy as a function of lattice spacing with energy difference E′ in tensile strained state that facilitates anion insertion. (**c**) Scheme representing the unstrained, strained, and recovered states of the NiTi alloy and the transferred strains on the surface oxide resulting in redox potential shifts. (**d**) Potential well representation of the transition between these states for electrochemical processes with E′ for the strained state schematically illustrated in panel (**b**).

To characterize the effect of quantifiable strain on electrochemical performance, we build on the principle that Ni-Ti-O based oxide is active for the redox reaction with OH^−^ ions in alkaline electrolyte solutions^33^,^51^,^52^. Due to the ability to strain set conductive NiTi alloys, this provides an excellent platform for characterizing the role of strain transferred to the active Ni-Ti-O containing surface oxide layer on the observed redox couple in alkaline electrolytes. Cyclic Voltammetry (CV) was carried out using 2M NaOH electrolyte in a 3-electrode configuration with a Saturated Calomel Electrode (SCE) reference and a platinum counter electrode at scan rates of 100 mV/s. As previous studies have demonstrated the stability of the SCE reference electrode to address the electrochemical response of NiO, Ni(OH)_2_, and Ni-Ti-O based metal oxides in alkaline solutions, it was chosen as the reference electrode for the electrochemical tests^51^,^53^,^54^,^55^. (Figure 3) For purposes of comparison, the current densities were normalized to analyze the potential shifts in the anodic and cathodic branches. To accurately measure the redox peak potentials and exclude other possible sources of errors, 100 CV sweeps at a scan rate of 100 mV/s were performed within the operating voltage window to obtain reproducible voltammograms. To ensure accuracy, Gaussian fits were applied to the anodic and cathodic peaks to measure the peak potentials. CV curves comparing redox performance for both (1) different amounts of total strain applied to the surface-bound NiO-TiO_2_ materials (Fig. 3a) and (2) the same materials except with the strain recovered in a manner consistent with Fig. 1c (Fig. 3b) elucidate the principle that strain has an evident and reversible effect on the voltage of the redox couple. In the first case, the shift of both the anodic and cathodic peaks toward lower potentials indicates that the observed effect is not explained by resistance or activation polarization in the electrode. The strain-induced modification to the redox energetics is further highlighted by experiments where the strain on the NiO-TiO_2_ based active material can be partially recovered, leading to a shift of both the anodic and cathodic peaks toward the native potential of the redox couple measured in the unstrained material. The observation that strain recovery of the (strained) active material leads to reversal of shifts in the electrochemical response associated with strain supports key ideas: (1) it mitigates the role of other phenomena in the electrochemical potential shifts, since the electrolytes, reference electrode, and testing conditions are otherwise invariant in these tests, and (2) supports the integrity of the NiTi-oxide interface, since delamination effects under strain applied to the NiTi would inhibit further strain transfer or recovery and would expose the NiTi material to the electrolyte leading to no measureable electrochemical material response. These results suggest for the first time that mechanical strain as an input parameter can modify or control electrochemical reduction potentials relevant to metal oxide based energy storage materials. Whereas previous recent reports have indicated the effect of strain on catalysis^16^,^17^,^56^,^57^,^58^, our results are both consistent with these reports, but with key differences. Similar to a catalytic system where charge-transfer reactions are confined to a surface, reduction and oxidation reactions contributing to the pseudocapacitance of NiO-TiO_2_ involves surface reaction with OH^−^ ion from the electrolyte. Previous studies have indicated that doping effects can modify the oxidation state changes of Ni-based metal oxides^59^,^60^,^61^ where equilibrium redox potential (*E*_*eq*_) shifts can be attributed to structural distortions from doping^59^. In our study, we demonstrate mechanical strain as an input parameter, verified by XRD and Raman spectroscopy, where the local distortions of the surface oxide facilitate insertion of the OH^−^ ion into the surface oxide layer. This is distinguished from shifts in *E*_*eq*_ due to electrolyte concentration, heating, or modification of reference potentials since strain application and strain recovery trigger and reverses shifts in *E*_*eq*_ while electrolyte, reference electrode, and testing conditions are invariant. This is enabled by taking advantage of the shapememory response of NiTi alloy to recover the structural distortions imposed by the applied tensile strain on the surface oxides, which in turn recovers the electrochemical response. In all cases, we observe that tensile strain lowers the equilibrium redox potential associated with the physical insertion of anions to store energy in the NiO-TiO_2_-based material in a manner that correlates with the total amount of applied strain (Fig. 4a and supporting information, Fig. S8). This change in reduction potential can be reversed by recovering the strain imposed in the material. As only elastic strains have the ability to simultaneously affect Raman modes, lattice spacing, and electrochemical behavior, the NiTi platform provides a versatile substrate for locking such strains in metal oxides deposited on the surface.

Based on these observations, we propose a simple concept to describe this effect that is illustrated in Fig. 4b. By applying tensile strain to the nanostructured material, the total free energy of the crystal is above its equilibrium value as described through relationships between the total cohesive energy of the crystal and the lattice parameter, such as the universal binding energy relationship (UBER). As only <0.1% strain is transferred to the surface oxide even though the alloy undergoes 15% tensile strain, the reversible effects observed in electrochemical measurements (Fig. 4c) originate from the changes in the energy landscape of the surface oxide. Since this process requires the insertion of an anionic species into the host lattice, accompanied by an oxidation state change in the metal oxide, the increase in free energy facilitates a smaller energy barrier between the strained metal oxide (A′) and the inserted state (B) in comparison to the unstrained metal oxide (A) and the inserted state (B). This is visually represented through a potential well diagram shown in Fig. 4d. This process is distinguished from that described in electrocatalytic reactions, since effects in such systems are likely to be minimal until the imposed strain is of a significant magnitude to modify the electronic band structure of the catalytic material. Even in the case of applied strain at <0.1%, our results indicate a marked effect of strain on electrochemical redox reactions relevant to redox-based energy storage systems that could be a critical tool in the future vision of engineered materials for advanced energy storage systems. Moreover, the strain can be applied to the surface oxide and be reversed to varying degrees based on the extent of preloading, thickness of the surface oxide, and anchoring to the NiTi surface which leads to isolation of mechano-electrochemical effects of energy storage redox active materials owing to the fact that the only parameter capable of reversing redox potential shifts based on this system are the mechanical strains present in the surface oxides. Also, transformational structural distortions that accompany ion insertion into metal oxide lattices during electrochemical reactions in pseudocapacitors and batteries can be studied using the NiTi platform. This would offer a control knob that can directly tailor energy landscape of existing energy storage materials.

## Conclusion

In summary, we demonstrate the ability to leverage strain engineering to modify the electrochemical potential of Ni-based metal oxide nanostructures fabricated on the surface of superelastic/shape memory NiTi materials during OH^−^ insertion and extraction. With less than 0.1% strain, we observe shifts in the electrochemical potential up to ~30 mV. Notably, this effect is uniquely correlated with strain as the reversal of strain in the material (a feature enabled by the superelastic NiTi) leads to a subsequent recovery of the electrochemical potential shifts. This elucidates the strain tensor as a six-dimensional framework to modify the electrochemical response of materials, opens a new area where foundational principles of electrochemistry (such as the Nernst potential) can intersect mechanical properties of materials, and provides a practical framework for improving the function of energy storage materials. As pairing of anodic and cathodic potentials dictate the total energy density of a battery, strain could potentially open a route to improve energy storage performance of batteries building from already existing materials, instead of engaging new synthesis driven routes toward new materials. Unlike semiconductor manufacturing routes that leverage strain engineering to modulate electronic properties of materials, we propose electrochemistry to be more amenable to strain engineering since both the electronic properties of a material and the physical insertion and storage of ions in a material are attributes that can be controlled with strain, as we discuss in this work.

## Experimental Methods

### Aging of NiTi superelastic wire

NiTi superelastic wires (0.5 mm diameter, 55% Ni from Nitinol Devices & Components, Inc.) were repeatedly sonicated for 10 min in Acetone (Aldrich) followed by Ethanol (Aldrich) and then nanopure water (Millipore water purifier). The wires were dried in air and were subjected to aging process by heating them to about 600 °C for 1 hour under vacuum with a small controlled flow of air. This led to the formation of a ~200 nm mixed oxide layer on the surface of the wires. The aging process was used to perform two functions; the activation of the superelastic/shapememory capability of the NiTi wire and to grow a thin oxide layer on the surface of the wire.

### Strain setting the surface oxide

The NiTi superelastic wires were subjected to tensile deformation up to 10% and 15% strains at a rate of 2 mm/min using an Instron 5944 mechanical testing system. The unstrained (0%) and the tensile deformed (10% and 15%) wires were subjected to sonication treatment for 30 min in 30% peroxide solution to impart nanotexturing on the oxide surface and to electrochemically activate the surface oxide layer. The treated wires were rinsed in nanopure water followed by drying in air.

### Differential Scanning Calorimetry

Differential Scanning Calorimetry (DSC, TA Instruments) was performed on the unstrained and strained wires to understand the strain recovery property of the alloy. The unstrained and strained wires were heated from room temperature to 100 °C in the first heating cycle followed by cooling to −100 °C in the cooling cycle and equilibrating at these respective temperatures for 5 min in aluminum pans. The subsequent heating cycle was from −100 °C to 100 °C followed by cooling. After two heating and cooling cycles the wires were equilibrated at room temperature.

### Electron Imaging

Characterization of the microstructure and Energy Dispersive Spectroscopy analysis was performed using a Zeiss Merlin SEM at various magnifications using 5 KV beam voltage for imaging and 20 KV beam voltage for EDS elemental analysis. Characterization of the nanostructure of the surface oxide was performed using FEI Tecnai Osiris TEM using a 200 kV S/TEM system. STEM EDS maps were obtained on an oxide flake scrapped off from the surface of the NiTi wire to further characterize the composition of the surface oxide.

### Raman and XRD characterization

Raman spectroscopy measurements were carried out using a Reinshaw Raman microscope using 532 nm Laser excitations. Maps comprising of 800–1000 spots across the surface of the wire was obtained with 60s exposure time at 10% laser power to yield a statistical strain distribution of the strained surface oxide layer. Mean peak shift corresponding to various Raman active modes of the surface oxide was obtained by using Lorrentzian fits on the obtained spectra. XRD measurements were carried out using a Scintag XGEN 4000 using Cu Kα 1.542 A°. To yield good X-Ray counts, 40 sec exposure times per 0.2 degree increments were maintained throughout the measurement.

### Electrochemical Measurements

Electrochemical measurements were performed using a 3 electrode configuration in a beaker type cell with the NiTi alloy with surface oxide as the working electrode, a platinum foil (Alfa Aesar 1 cm × 1 cm) as the counter electrode and a Saturated Calomel Electrode (SCE) as the reference electrode. The electrolyte used was a 2 M NaOH solution. Cyclic voltammograms were obtained for all the unstrained and strained states of the surface oxides at a scan rate of 100 mV/s in the voltage range of 0 V to 0.5 V. All the samples were cycled for a 100 cycles at 100 mV/s to get reproducible voltammograms. All electrochemical data was normalized to the immersed area of the wire electrodes.

### Strain Recovery and Analysis

The strain recovery was performed by heating the strained wires with strained surface oxides to 60 °C in a Quartz CVD tube furnace under vacuum for 1min. Due to the recovery process the strain imposed on the wire and the oxide was recovered. The change in the wire length after the recovery process was used to estimate the strain recovery by the NiTi alloy. Raman and XRD measurements were performed on the strain recovered to study the recovered strain state of the surface oxide followed by electrochemical testing using the same conditions as the unstrained and strained wires.

## Additional Information

**How to cite this article**: Muralidharan, N. *et al*. Strain Engineering to Modify the Electrochemistry of Energy Storage Electrodes. *Sci. Rep.* **6**, 27542; doi: 10.1038/srep27542 (2016).

## Supplementary Material

Supplementary Information
