# Supplementary material for: Strain Engineering to Modify the Electrochemistry of Energy Storage Electrodes
Source: Sci Rep. 2016 Jun 10;6:27542. doi: 10.1038/srep27542 (PMC4901311; doi:10.1038/srep27542)
Supplement: Supplementary Information [file srep27542-s1.doc]

Supplementary Information

Strain Engineering to Modify the Electrochemistry of Energy Storage Electrodes

*Nitin Muralidharan^1^, Rachel Carter^2^, Landon Oakes^1^, Adam P Cohn^2^ and Cary L. Pint^1,2,*^*

^1^Interdisciplinary Materials Science Program, Vanderbilt University, Nashville, TN 37235

^2^Department of Mechanical Engineering, Vanderbilt University, Nashville, TN 37235

**SEM micrographs of tensile strained and recovered wires**


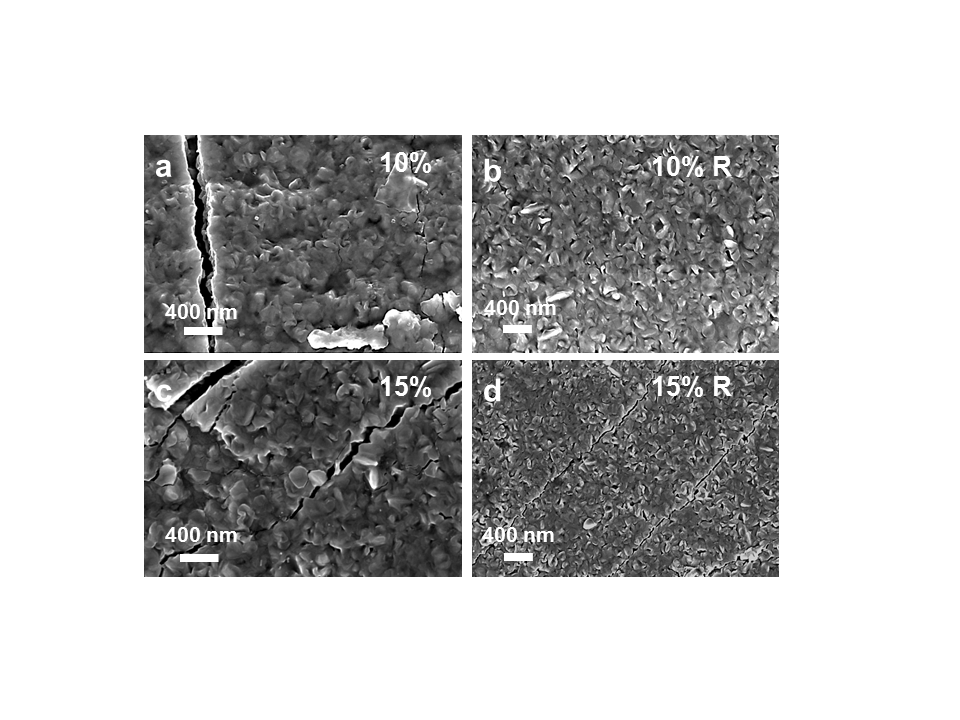


**Figure S1.** SEM micrographs of the nanotextured surface oxides at a) 10% tensile strained, b) 10% strain recovered, c) 15% tensile strained and d) 15% strain recovered

Figure S1 (a-d) show the scanning electron micrographs of the strained and strain recovered wires representing the strained 10%, 15% and recovered 10% R and 15% R. The nanotextured oxide undergoes cracking during the straining process which represents areas of strain release which lowers the overall strain transfer from the alloy to the surface oxide even if the intimate contact of the as grown oxide on the wire is strong. The NiTi alloy is superelastic whereas the ceramic based NiO-TiO_2_ oxide is brittle; cracking of the surface oxide is possible at very low strains of about 1% according to previous reports.^1-3^ Upon recovery, the cracked oxide appears to almost revert back to the unstrained state in the case of 10% R whereas cracking is still observed in 15% R which indicates a better strain recovery in the case of 10% R which is consistent with the observations from mechanical tests.

**EDS map of cross-section of the wire with surface oxide**


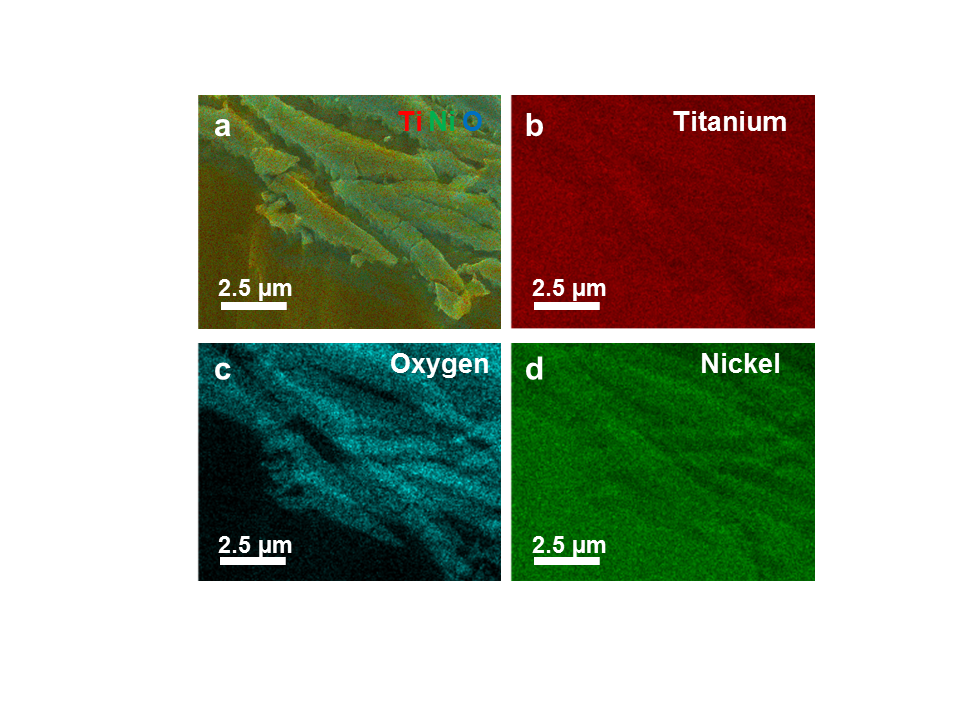


**Figure S2.** EDS maps of cross-section of the wire with the strained surface oxide; a) Full EDS map showing Ni, Ti and O distribution, b) EDS map showing Titanium distribution, c) EDS map showing Oxygen distribution and d) EDS map showing nickel distribution.

Figure S2 (a-d) shows the EDS maps of the cross-section of the NiTi wire representing Titanium, Oxygen and Nickel elemental distributions. The cracks on the surface oxide are partly due to the strain imposed on the wire and partly due to the mechanical cutting process of the wire. EDS maps suggest the surface oxide is composed of a mixed Ni-Ti-O based surface oxide. Cross-sectional image analysis show the thickness of the surface oxide is about 200nm. Figure S2 C shows the EDS map of Oxygen distribution indicating low oxygen concentration in the bulk alloy and in-between the cracks of the surface oxide.

**EDS Spectra of cross-section of the wire with surface oxide with compositional analysis**


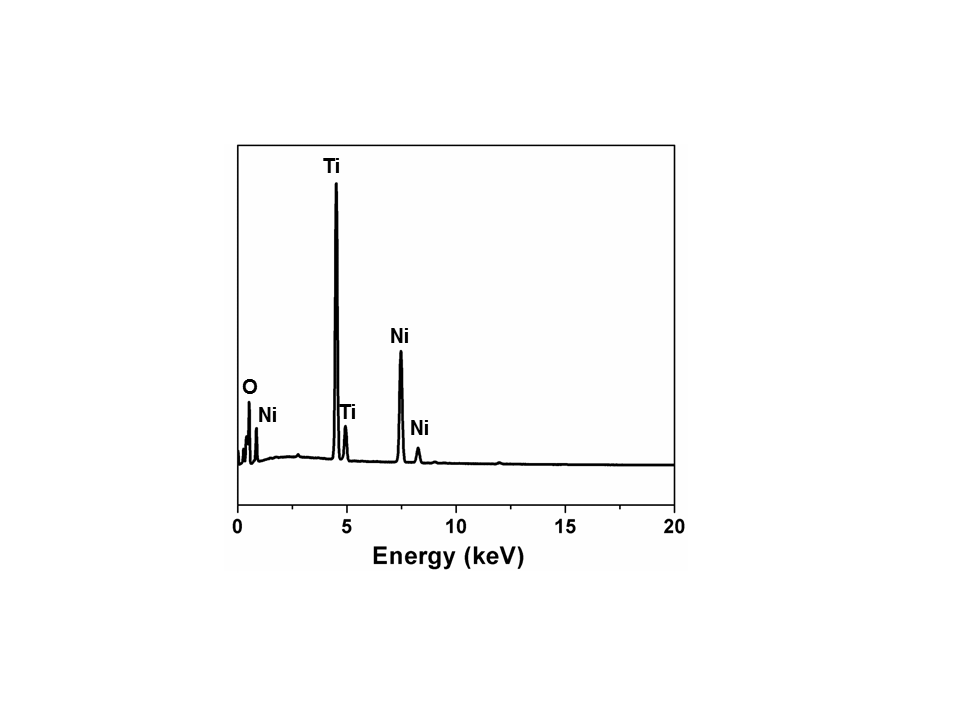


**Figure S3.** EDS Spectra of cross-section of the wire with surface oxide

EDS spectra shown in Figrue S3 indicates the presence of a Ni-Ti-O based surface oxide which comprises of a mixed oxide phase consisting NiO, TiO_2_ and NiTiO_3_ layers based on Raman and XRD analysis.


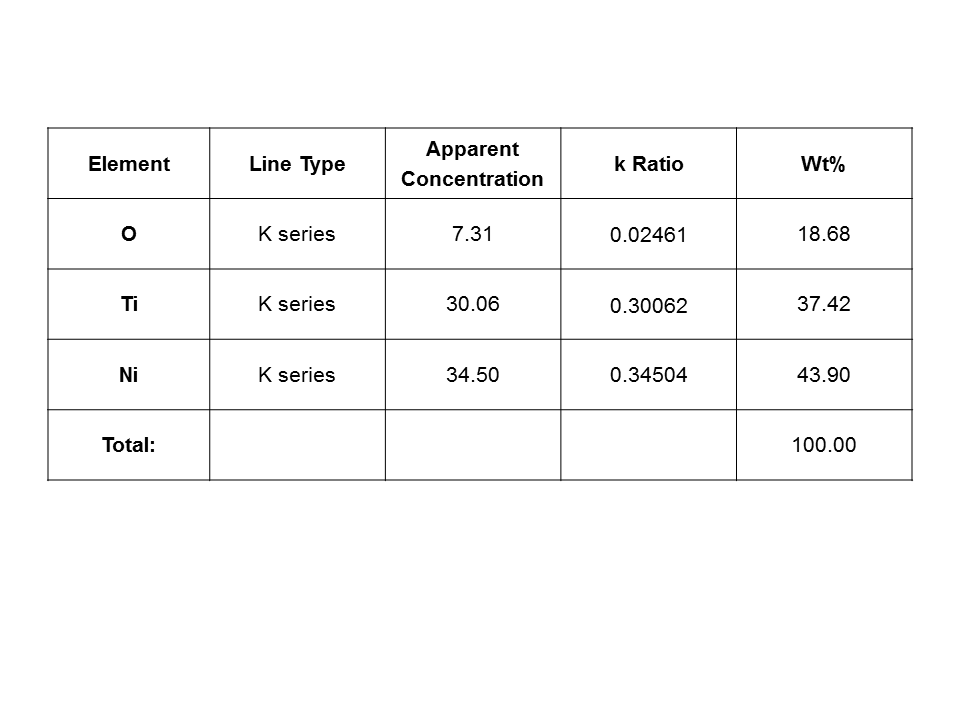


**Figure S4.** Compositional analysis based on EDS spectra of the cross section of the NiTi wire with surface oxide.


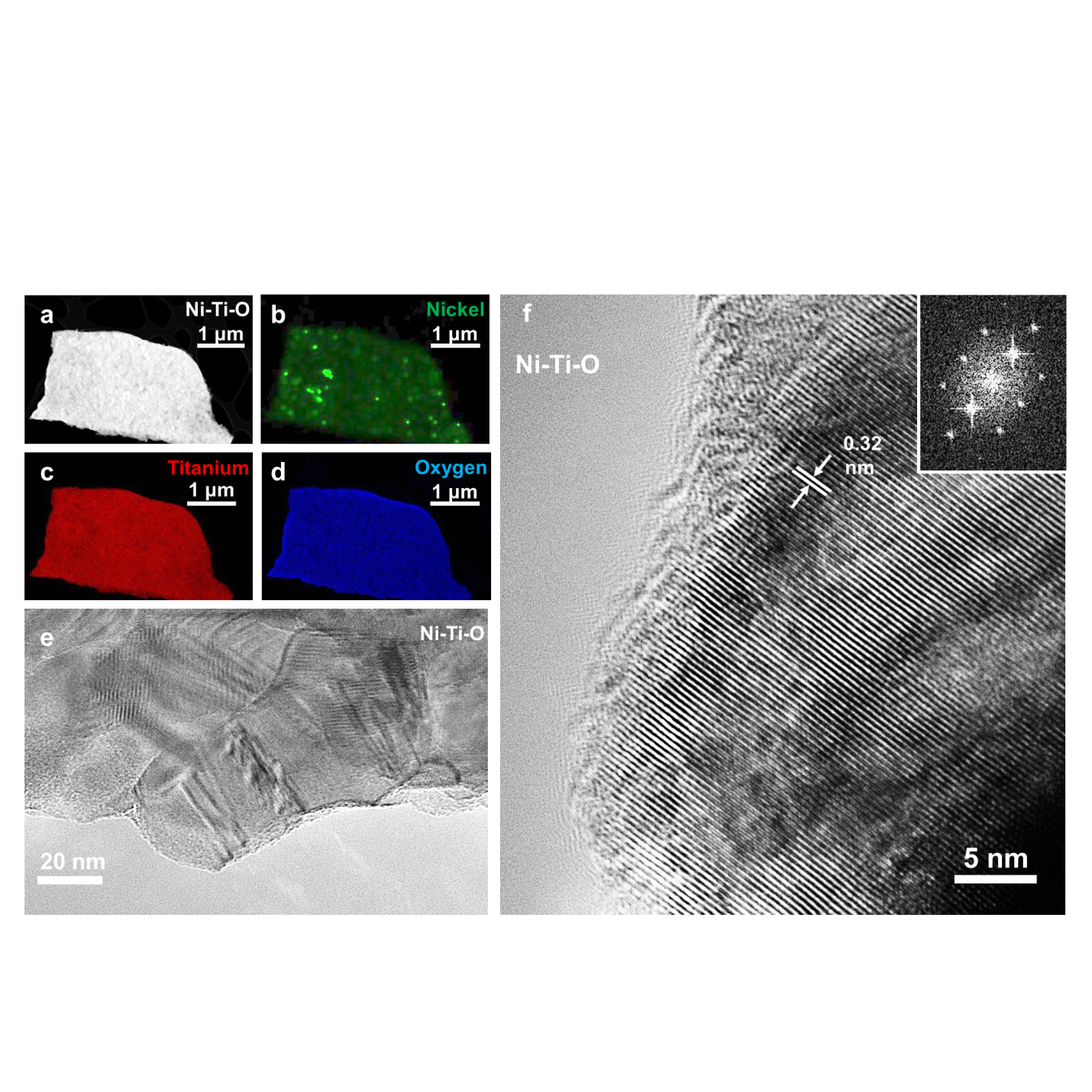


Figure S5. a) HAADF image of a Ni-Ti-O surface oxide flake, (b-d) STEM EDS maps of the surface oxide showing elemental composition of Nickel, Titanium and Oxygen, c) HRTEM image of the nanostructured surface oxide showing a polycrystalline surface oxide and d) HRTEM image of the surface oxide showing well defined crystal lattice, inset: diffraction pattern of the crystal lattice after performing FFT analysis.

TEM analysis was performed on the surface oxide by scratching off oxide from the wire surface on to a copper grid. As the oxide is adhered to the surface of the wire, scratching the surface results in further fragmentation of the oxide. The elemental composition of the surface oxide was observed using HAADF and STEM EDS maps (Figure S5. a-d). The presence of a polycrystalline surface oxide feature was observed through HRTEM micrograph (Figure S5e). At higher magnifications, clear distinguished crystal lattice of the surface metal oxide was observed (Figure S5f). FFT analysis was performed on the crystal lattice to determine a spacing of 0.32 nm between the observed atomic planes.

**XRD full spectra**


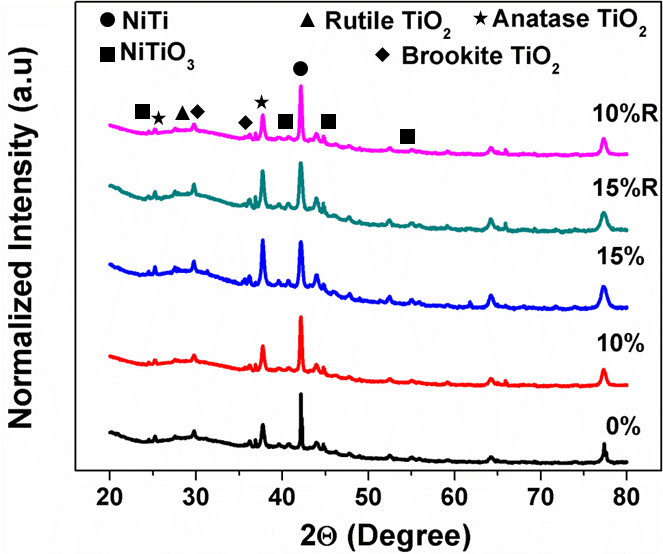


**Figure S6.** XRD full spectra of the surface oxides on NiTi alloy

X-Ray Diffractograms of the unstrained (0%), strained (10% and 15%) and recovered (10% R and 15% R) states of the wires and surface oxides were obtained using -Ray diffraction (Scintag XGEN 4000) using Cu Kα 1.542 A^o^. As the thickness of the wire was 0.5 mm, signal acquisition was performed between 2θ values of 20^o^ and 80^o^ with 40 sec per 0.2^o^ exposure time. Due to the geometry of the wire, tensile strain along the axis of the wire is accompanied by a reduction in thickness along the radial directions of the wire and on recovery this is reversed causing a possibility for biaxial effects of strain on the surface oxide. The peak positions were indexed using JCPDF cards 29-1360, 21-1272, 21-1276, 33-0960 and 18-0899. As only elastic strains on the oxide could result in such peak shifts, the % of elastic strain on the oxide on the different planes of the surface oxides were determined using the equation 1:

% elastic strain =
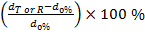
 (1)

where *d_T_,* *d_R_* and *d_0%_* represent the *d*-spacing of the tensile strained states, the recovered states and the unstrained (0%) strained states respectively. In all cases, only a small percentage of strain is transferred to the surface oxides even though the strain applied and recovered on the NiTi alloy is large. As the ceramic oxide layer on the surface is brittle, this is an expected result.

**Cyclic voltammograms of tensile strained and recovered states without current density normalization**


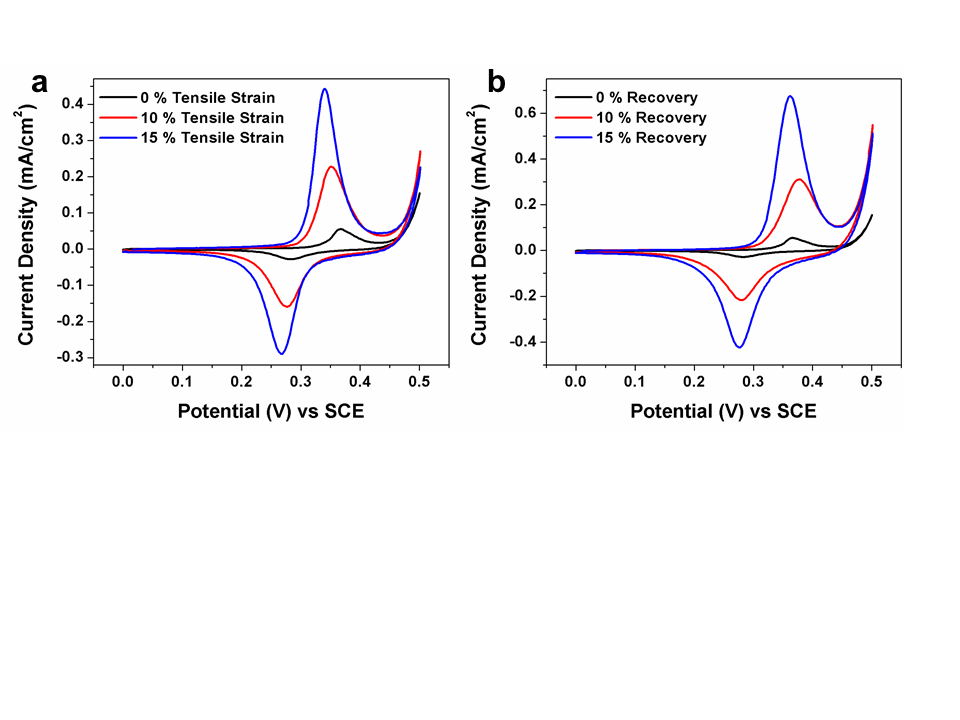


**Figure S7.** a) Cyclic voltammograms of the tensile strained states compared to the unstrained state and b) Cyclic voltammograms of the strain recovered states compared to the unstrained state.

Figure S7 (a and b) show the cyclic voltammograms of the unstrained, strained, and strain recovered surface oxides. Based on the EDS spectra, it is evident that the surface oxide is composed of Nickel and Titanium oxides. Based on the XRD strain analysis and Raman spectra, there is evidence of strain on the surface oxides which show patterns with the behavior of the NiTi alloy. The cumulative effect of these imposed and recovered surface oxide strains on the electrochemical performance of the NiO-TiO_2_ based surface oxide was examined using Cyclic Voltammetry using 2M NaOH electrolyte in a 3-electrode configuration with a Platinum foil (1cm X 1cm, Alfa Aesar) and a Saturated Calomel Electrode (SCE) as the counter and the reference electrodes. The potential is measured against an SCE electrode in a 3-electrode configuration which enables accurate characterization of the redox potentials. The NiTi wires with different strain states with nanotextured surface oxide were CV cycled 100 times at a scan rate of 100 mV/s to obtain reproducible voltammograms. Based on the redox potentials vs SCE, and redox peak potentials based on previous reports,^4, 5^ the surface redox process is attributed to a near surface reaction corresponding to the insertion and extraction of OH^-^ ions from the alkaline electrolyte, leading to a modification of the oxidation state of the active Ni-Ti-O material. Prior to all measurements, peroxide sonication step was employed to impart nanotexturing to the surface oxide and to electrochemically activate the surface for both strained and unstrained states of the alloy.


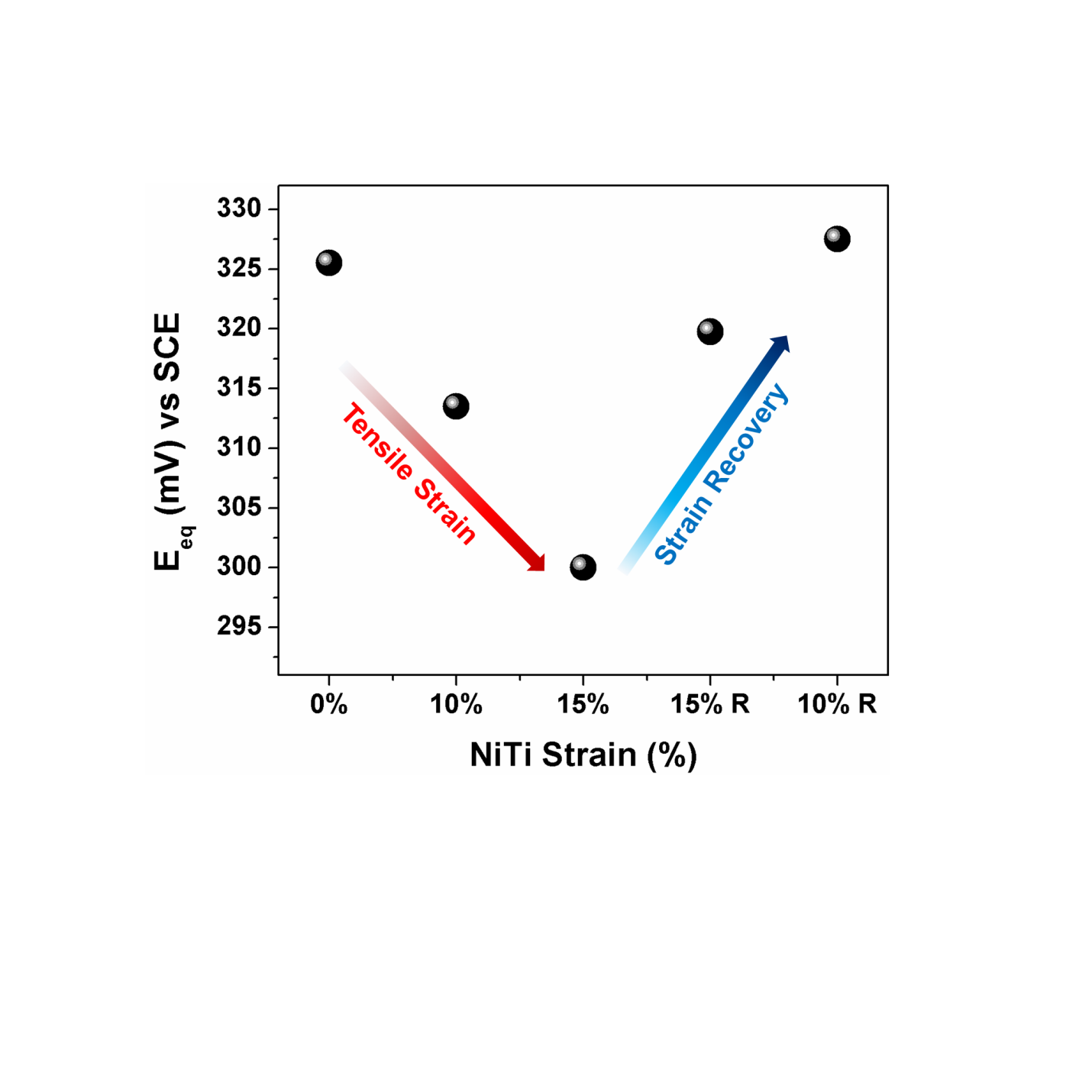


Figure S8. Variation of equilibrium redox potential with applied and recovered strain conditions.

The equilibrium redox potential *E_eq_* or *E_o_* is given by the equation,


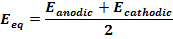


where, *E_anodic_* and *E_cathodic_* are the anodic and cathodic peak potentials shift corresponding to the unstrained, strained, and recovered states. The shift in the *E_eq_* or *E_o_* values with applied strain and recovered strain conditions is a consequence of the changes occurring to the crystal lattice of the redox active metal oxide. This effect is otherwise observed in doped metal oxides where slight distortions (strains ~ <1%) through doping alters the energy landscape of the redox active species. As the process of electrochemical insertion and extraction of ions during the redox reaction is accompanied by oxidation state changes to the redox active species, structural distortions are often a negative consequence. However, through the effect of metal ion doping in to metal oxide lattices, these distortions can be minimized resulting in easier insertion and extraction of electrolyte ions which leads to changing equilibrium redox potentials. In our study, we use small lattice strains (< 1%) to incur the same type of shifts in the equilibrium redox potentials and reversing the shifts through the strain recovery process.

REFERENCES

1. Undisz, A.; Schrempel, F.; Wesch, W.; Rettenmayr, M. *J. Biomed. Mater. Res. Part A.* 2009, 88, 1000-1009.

2. Racek, J.; Šittner, P.; Heller, L.; Pilch, J.; Petrenec, M.; Sedlák, P. *J. Mater. Eng. Perform.* 2014, 23, 2659-2668.

3. Racek, J.; Stora, M.; Šittner, P.; Heller, L.; Kopeček, J.; Petrenec, M. *Shape Memory and Superelasticity* 2015, 1, 204-230.

4. Danczuk, M.; Nunes Jr, C. V.; Araki, K.; Anaissi, F. J. *J. Solid State Electrochem.* 2014, 18, 2279-2287.

5. Hang, R.; Liu, Y.; Zhao, L.; Gao, A.; Bai, L.; Huang, X.; Zhang, X.; Tang, B.; Chu, P. K. *Sci. Rep.* 2014, 4, 7547
